# Tensiomyography method used for neuromuscular assessment of muscle training

**DOI:** 10.1186/1743-0003-10-67

**Published:** 2013-07-03

**Authors:** Ligia D Rusu, Germina GH Cosma, Sorina M Cernaianu, Mihnea N Marin, Petre Florinel A Rusu, Daniel P Ciocănescu, Florin N Neferu

**Affiliations:** 1 Sports Medicine and Kinesiology Department, University of Craiova, Craiova, Romania; 2 Methodic and Theory Department, University of Craiova, Craiova, Romania; 3 Applied Mechanics, University of Craiova, Craiova, Romania; 4 University C. Brancusi, Tg. Jiu, Romania

**Keywords:** Assessment, Contraction, Muscle, Neuromuscular, Tensiomyography, Training

## Abstract

**Background:**

Within the structure of the skeletal muscle, there are fascicles of muscular fibers that are made up of serially distributed contractile elements. These elements are controlled by the nervous system, control which results in obtaining the muscular strength required for movement and its control. This study presents the neuromuscular assessment using tensiomyography method (TMG).

**Methods:**

We studied two groups of soccer junior players, group 1 (experimental group) and group 2 (control group), each containing 15 soccer players; we have considered two situations of muscle training: the combination between the isometric-concentric contraction for group 1 and the concentric contraction for group 2. TMG is the electrical stimulation of the muscle group and the recording of the muscle parameters resulting after the isometric contraction: time contraction (Tc) and displacement (Dm) at rectus femoris muscle (RF), pointing out two moments T1 and T2.

**Results:**

Tc decreasing and the Dm increasing involve a good response following the muscle training. For group 1, the Tc evolution is 22.54 ms/22.45 ms (T1/T2) for the right RF and 22.65 ms/22.26 ms for the left RF, while for group 2 results in a Tc evolution of 24.33 ms/28.57 ms (T1/T2) for the right RF and 25.74 ms/28.61 ms for the left RF. Dm for group 1, results in a 6.57 mm/6.85 mm (T1/T2) for the right RF and 6.92 mm/7.06 mm for the left RF, while for group 2, the Dm evolution shows 7.45 mm/5.83 mm (T1/T) for the right RF and 7.41 mm/6.26 mm for the left RF. Also, the evaluation on motor test indicated better results on T2 for the experimental group. Summarizing the results of Student t-test, we found significant differences between the averages of the two groups in all parameters (p < 0.001), the experimental group registering better results than the control one.

**Conclusions:**

It is possible to develop muscle training which can be monitored through TMG.

## Background

Considering the field-related studies [1], it has been assumed, so far, that there is a correlation between the percent of muscle fibres of the type I or II and athletic performance, according to the exercise level and the athletic skills of the individuals. The role of this study is to present the importance of muscle fibre evaluation applying a non-invasive method for neuromuscular evaluation and show the importance in monitoring the muscle training.

From this point of view we consider that the assessment of muscle fibre structure indicates the correlation between the muscle contraction, following the stimulation, and the muscle fibre structure, the proportion in which each of these fibers is represented, varying with muscle activity. Apart from these types of muscular fibers, there are also type IIc muscle fibers, usually considered intermediate fibers that have a high potential to turn into type I, IIa or IIb fibers; this transformation is supposed to happen during the regeneration period, this phenomenon representing muscle plasticity. This structural diversity is the result of the description of a large number of phenotypes, having a functional significance which remains unclear [2]. The factors that influence these variable structural aspects could be genetic programs, hormonal influences, weariness (particularly important from the point of view of functional recovery). The total number of striated muscular fibers cannot change; only the percentage of muscular fibers (fast and slow twitch) within skeletal muscles can vary and this is influenced by muscular plasticity, as previously mentioned [3]. This property of skeletal muscle is affected in the pathological processes that regard either the nervous or the neuroendocrine, muscular or vascular systems; this influence is expressed within the muscular fiber metabolism. The transformation of the muscular fiber metabolism is an aspect that can be controlled through a complex medical intervention, following complex evaluation of the structure and contractile properties of the muscular fibers [4].

The functional features of the muscle depend on the presence of the myosin heavy chain (MyHC), on the mechanical task and also on the role of the muscle group. The speed of the muscle contraction also represents a marker for muscle tissue qualities.

The skeletal muscle includes two types of muscle fibres, as well as, two types of motor units (MU) Type I, IIa, IIb (x). During muscle contraction, the MU is progressively recruited according to the muscle activity. So during maximal effort, the recruited MU are Type I-Type IIa-Type IIx. Understanding the structure of muscle fibres, as well as that of the recruited MU, represents an aspect which influences the muscle force. Muscle force depends on the number, the type of motor units and on the frequency of muscle stimulus. So the MU Type II generates a higher level of muscle force due to a larger volume of muscle fibres, as compared to type I fibres.

An increase in muscle contraction speed involves a decrease in muscle force. This aspect is more important and more visible during concentric muscle contraction, when dealing with eccentric muscle contraction, a maximum muscle force is delivered.

Type I of the muscle fibres display resistance to muscle fatigue and they are involved in the endurance effort due to their oxidative metabolism. Type II muscle fibres have an anaerobe metabolism and no resistance to muscle fatigue. They are first recruited during maximal effort. There are numerous questions and discussions regarding the role of Type IIx muscle fibres, but most of the authors approach only the role of the transition from Type I to Type II and vice versa.

Tensiomyography (TMG) is an evaluation method for the morphofunctional potential of the muscle, which allows the detection of the muscular reaction to electrical stimulation. Through this method we can appreciate the ratio between type I (fatigue-resistant) and type II (white, fast-twitch, with low resistance to fatigue – this phenomenon appearing before the completion of the electrical stimulation process) muscular fibres.

## Materials and methods

### Study design

TMG is a non-invasive method which determines the diagnosis of a certain muscular type (types of muscular fibres) and muscular status/condition (fatigue, stress influence on the body, etc.), the diagnosis of a functional muscular symmetry [5,6], either temporal or morphological, the evaluation of muscular synchronization, fast detection of an infra-clinical lesion of the muscle in situ (less than 5 minutes).

**Figure 1:**
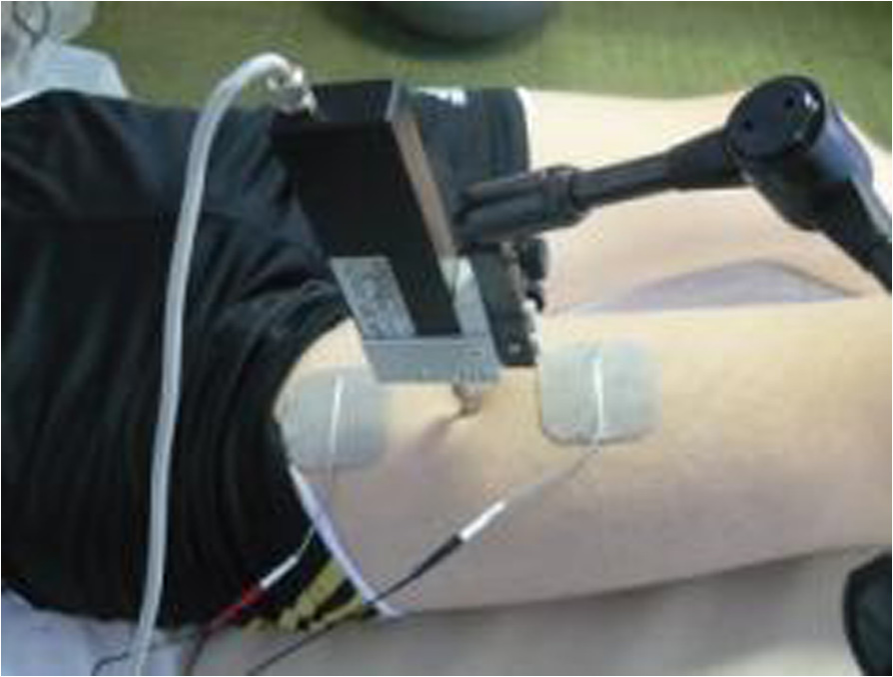
Placement of TMG sensor.

TMG also demonstrates a connection between the twitch contraction time of the entire muscle and the percentage determined histochemically in the muscular fibres with slow contraction. The evaluation of muscular training can be made under intermittent electrical stimulation of the muscle. This stimulation is made with a TMG–S1 electro-stimulator (Furlan & Co., Ltd.), using 5/5 cm Platinum-type electrodes. The stimulation is performed under increasing electrical current intensities, between 10–65 mA, the length of the stimulation being one millisecond. An isometric contraction is generated by the electrical stimulation. The detection of the muscular response to the electrical stimulus is performed with a G40, RLS Inc. sensor, perpendicular to the muscle surface, in the area in which the muscular geography is well displayed (this can be more precisely determined if the subject is requested to perform an isotonic contraction, if a muscle strength higher than 2 is possible). The sensor is placed at this level; it will exert a 0.7 N/mm2 pressure on the contact surface. This pressure is called pretension [7] and its role is to increase the response to the applied electrical stimulus. Due to the electrical stimulation, a transversal movement of the muscular fibres will occur and the sensor will record this. The amplitude of this transversal movement is proportional with the muscular force and the percentage of type I muscle fibres. The measurement of the muscular response, the data storage and the analysis, have been made with dedicated TMG software. The investigation has been performed to the level of the rectus femoris (RF) on both sides, right and left (Figure 1). The soccer athlete is in prone position, knee flexion 10^0^ and sustained by a support and the sensor is placed under the maximal point of contraction detected by the isometric voluntary contraction. This topographic point is the place where the TMG sensor will be fixed.

We specify that the sensor which receives the muscular response to stimulation is placed in the maximum contraction point, and the electrodes are placed on both sides of the sensor.

### TMG recording

The TMG signals are received by a Matlab Compiler Toolbox on a 1 kHz frequency. Two supra-maximal responses are stored and then the average is calculated. The supra-maximal stimulation [5] is regarded as corresponding to a minimal stimulation and it determines maximum amplitude of muscular deformation, recorded as Dm.

The parameters evaluated through TMG were:

•Contraction time (Tc) the time between the moment when the muscular contraction is 10% and the moment when the contraction reaches 90% out of maximum (ms). The value of the contraction time depends on the percent of fast or slow fibres [8] at the level of the studied muscle. Thus, the values decrease once the percent of type II fibres increases, and they increase when the type II fibre percent is low and when that of type I fibres is high.

•The amplitude of muscular displacement in transverse direction- Dm (mm) is a parameter which is also correlated with Tc values and depends on the flexibility of muscular tissue. Therefore, Dm values increase when the explosive force is developed, involving high movement amplitude, and they decrease under the conditions of a high muscular tone.

The two parameters enable us to appreciate the muscular composition of the studied muscular groups [8], correlated with the increase of contraction time and the decrease of muscular displacement amplitude [9]. These parameters have normal average values for Tc, namely, 32.83 ms and the average Dm value is 8.17 mm for all muscular groups. We mention that Dm is a parameter whose diminution is associated to the increase of Tc and muscle tone.

The motor tests applied for confirming the results of the TMG neuromuscular investigation have included motor trials which evaluate the explosive force developed at the level of the inferior limbs. For this reason, we have used three different motor trials.

Standing long jump (the lower limbs strength test) Position of tiptoe is behind the jump line, each soccer athlete jumps through upper limb balance. We measure the distance between the jump line and the heel point contact after jumping, taking into account the best jump in two attempts.

The second motor test is:

Successive Jumps on Steps on the stairs of the stadium the soccer player jumps from two to two stairs along 10 m’s distance, in height. We perform two times tests and we note the best time of the test.

Remote Ball Hitting we outline a 10 m’s width corridor, the ball being placed on one of its limits. The subject strongly hits the ball three times with each foot, considering his/her best shot. The distance between the point where the ball is hit and the point where the ball touches the ground, inside the corridor area, is carefully measured. All recorded values have been considered for both inferior limbs, mentioning the dominant performance.

### Participants

We have submitted to the study two groups of junior soccer players, each group including 15 individuals, aged, on an average, 16 years (±0.4 months), male, weight 52 kg, height 170 cm, BMI-18, juniors level.

Group 1 - the experimental group and group 2 - the control group.

We make two assessments at the moment T1 and T2 involving both groups, before and after the specific muscle training, [10] which included the concentric contraction for group 2 and a combination of two contractions, isometric-concentric contractions, for group 1. One may notice that these methods represent the best ways for assessing muscle training.

All procedures used for this study were completed in accordance with the Declaration of Helsinki and were approved by the Committee for Human Experimentation within the Sports Medicine and Kinesiology Deparment and Ethics Committee of University of Craiova. For each participant we complet the informed consent according to the ethical regulations and after we present the methodologies of evaluation.

The training program has been expressly made up for each of the two groups of sportsmen.

The programs designed for the control group include exercises for strength development, focused on methods of concentric unique muscle contractions and those designed for the experiment group simply systematic techniques relying on combinations of two types of muscle contractions – isometric-concentric contractions.

#### Experimental group (group 1)

Each program consists of 3 sessions of exercises adjusted to the main purpose intended for the training phase:

First program for increasing the muscle mass; the training period general physical training phase (3 weeks); the working programs have involved exercises focused on isometric-concentric contractions ordered into 4–6 series of contractions, counting 6 limbering ups (about 40% -50% of 1RM, 4-5 s uphold), and an active break of 3 minute after each series.

Second program for maximum force development; the training period pre-competition stage 1 (3 weeks); the working programs have involved exercises focused on isometric-concentric contractions ordered into 3–4, counting 6 rehearsals (about 70% -80% of 1RM, 10s uphold), and a passive break of 3 minute after each series.

#### Control group (group 2)

- first program for increasing the muscle mass; the training period general physical training phase (about 3 weeks); the working programs have implied exercises focused on types of concentric contractions, grouped on series of 4–6 contractions, including a number of 6–8 limbering ups (70-80% of 1RM,), with a 3 minute passive break.

- second program for maximum force development; the training period pre-competition stage 1 (3 weeks); the working programs have involved exercises focused on concentric contractions grouped on series of 4, with a number of 4 rehearsals (80-85% of 1RM,), including a 5-minute active break.

The validation of methods relying on neuromuscular investigation has been achieved by means of motor tests specific to sports training; these tests express the speed of movement execution as a result of changes on the structure of muscle content, leading to the increase of the fast muscle fiber rate.

For all parameters the Student t-test was applied to see if the averages of the two groups are significantly different. Average and standard deviation have been calculated for all the variables. The post-hoc statistical power analysis for the two independent samples t-test was performed.

## Results and discussions

**Figure 2:**
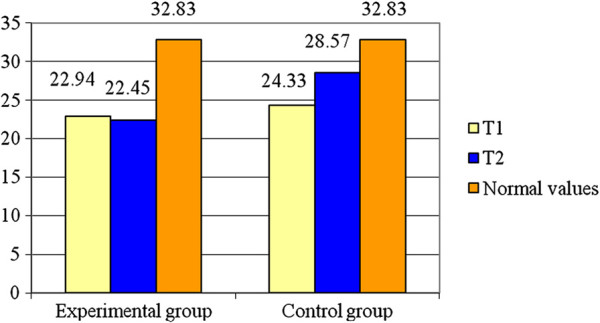
**Averages for T**_**c **_**– RF – R.**

Further on, we shall present the results of our assessment for Tc. Table 1 shows the statistical parameters for rectus femoris concerning the Tc and the difference between the time intervals, T1 and T2, for the same parameter, involving both groups, at the level of the right side of RF. Figure 2 reveals this evolution for the RF right side.

**Table 1:** **Statistical parameters for T**_**c **_**and D**_**m**_

**Parameters**	**Statistic parameters**	**G**_**1**_				**G**_**2**_			
		**T**_**1**_	**T**_**2**_	**Dif.**	**Dif. (%)**	**T**_**1**_	**T**_**2**_	**Dif.**	**Dif. (%)**
TC – RF - R	AVERAGE	22,94	22,45	−0,49	−2,14	24,33	28,57	4,24	17,43
	STDEV	2,08	2,14	-	-	3,8	3,28	-	-
TC – RF - L	AVERAGE	22,65	22,26	−0,39	−1,72	25,74	28,61	2,87	11,15
	STDEV	2,16	2,09	-	-	3,52	2,88	-	-
DM – RF - R	AVERAGE	6,57	6,85	0,28	4,26	7,45	5,83	−1,62	−21,74
	STDEV	2,8	2,72	-	-	1,14	1,46	-	-
DM – RF - L	AVERAGE	6,92	7,06	0,14	2,02	7,41	6,26	−1,15	−15,52
	STDEV	2,46	2,4	-	-	1,16	1,51	-	-

As can be seen from Table 1, the experimental group registers a decrease of about 2.14% (0.49 ms) from the initial testing while control group had an increase in Tc of about 17.43% (4.24 ms). The variable is normally distributed (p > 0.05). The Student test illustrates there is a significant difference between the two groups, for the RF right side (t = 6.06; p < 0.001). The value of statistical power of the test is 0.99 so the probability of obtaining a statistically significant result is high.

**Figure 3:**
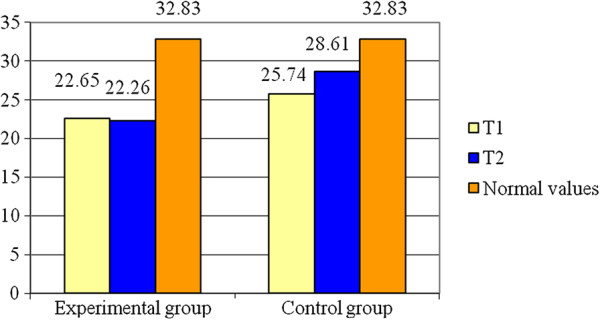
**Averages for T**_**c **_**– RF - L.**

Recording the RF-L, the Table 1 shows the statistical parameters concerning the Tc and the Figure 3 shows the evolution of the average values of this parameter. The obtained results show that the experimental group has a decrease of about 1.72% (0.39 ms) at the moment T2, while the control group presents an increase of about 11.15% (2.87 ms) at the same moment of the assessment. The variable is normally distributed (p > 0.05). The Student test shows significant difference between the average values for the two groups (t = 6.92; p < 0.001) for RF-L. The statistical power of the test (0.99) conducts to the probability of obtaining a high statistically significant result.

Bellow, we present the results regarding the displacement Dm, for rectus femoris.

**Figure 4:**
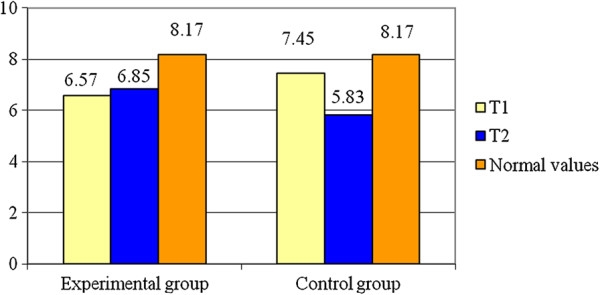
**Averages for D**_**m **_**– RF – R.**

In the Table 1, one may notice the results for RF-R. As well, the Figure 4 represents the values of Dm for RF-R at the moments T1, T2 as compared to the standard value. For the experimental group Dm increases with about 4.26% (0.28 mm) at the moment T2 and Dm of the control group decreases with about 21.74% (1.62 mm). The variable is normally distributed (p > 0.05). Also, statistical differences of means are identified between the two groups (t = 3.5; p < 0.05) for this parameter. The value of statistical power of the test is 0.23 so there is a higher probability for error than correct decision.

**Figure 5:**
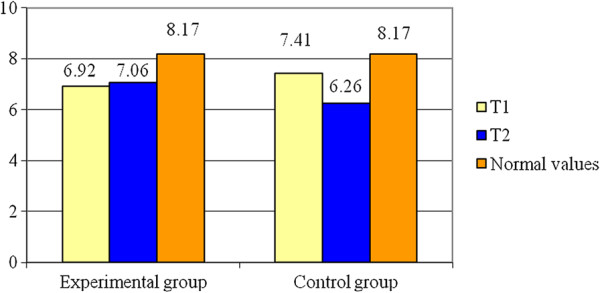
**Averages for D**_**m **_**– RF - L.**

The Dm values for RF-L are presented in Table 1 as well as in Figure 5. The variable is normally distributed (p > 0.05). Analysing the means of the two groups at the end of the study period we observe that there are significant differences between them (t = 2.52; p < 0.05). The statistical power of the test (0.18) conducts to a higher probability for error than correct decision.

**Figure 6:**
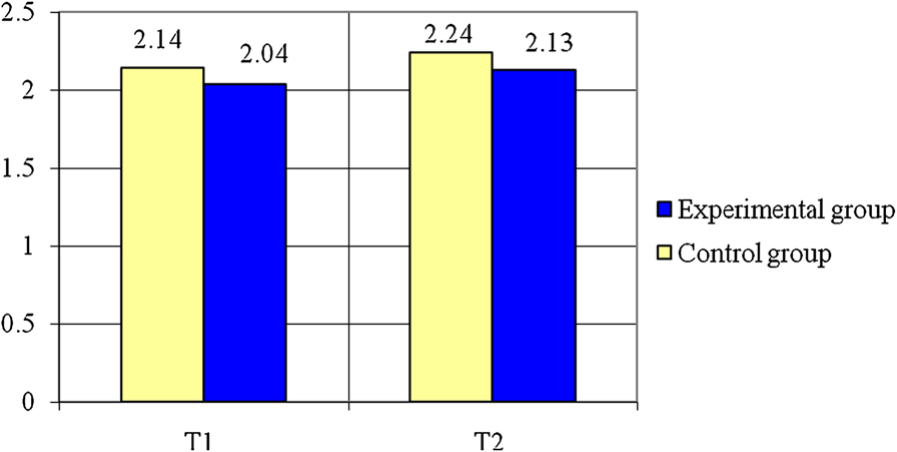
Standing long jump (m) – averages.

Concerning the Standing Long Jump (see Table 2, Figure 6) the experimental group registers an increase of 4.67% (0.1 m) to the final test as compared to the initial one while the control group had an increase of 4.41% (0.09 m) at the end of the study period. The variable is normally distributed (p > 0.05). The results of Student test show there are significant differences between the means of the two groups (t = 2.8, p < 0.05). The value of statistical power of the test (0.72) is considered acceptable.

**Table 2:** Statistical parameters for motor tests

**Parameters**	**Statistic parameters**	**G**_**1**_				**G**_**2**_			
		**T**_**1**_	**T**_**2**_	**Dif.**	**Dif. (%)**	**T**_**1**_	**T**_**2**_	**Dif.**	**Dif. (%)**
Standing long jump	AVERAGE	2,14	2,24	0,10	4,67	2,04	2,13	0,09	4,41
	STDEV	0,11	0,08	-	-	0,16	0,14	-	-
Successive jumps on steps	AVERAGE	2,67	2,51	−0,16	−5,99	2,78	2,65	−0,13	−4,68
	STDEV	0,15	0,14	-	-	0,13	0,15	-	-
Remote ball hitting - dominant inferior limb	AVERAGE	42,80	48,53	5,73	13,39	39,47	43,87	4,4	11,15
	STDEV	5,7	6,01	-	-	5,96	5,3	-	-
Remote ball hitting - non-dominant inferior limb	AVERAGE	24,87	29,53	4,66	18,74	23,13	26,87	3,74	16,17
	STDEV	4,67	3,7	-	-	3,42	2,85	-	-

**Figure 7:**
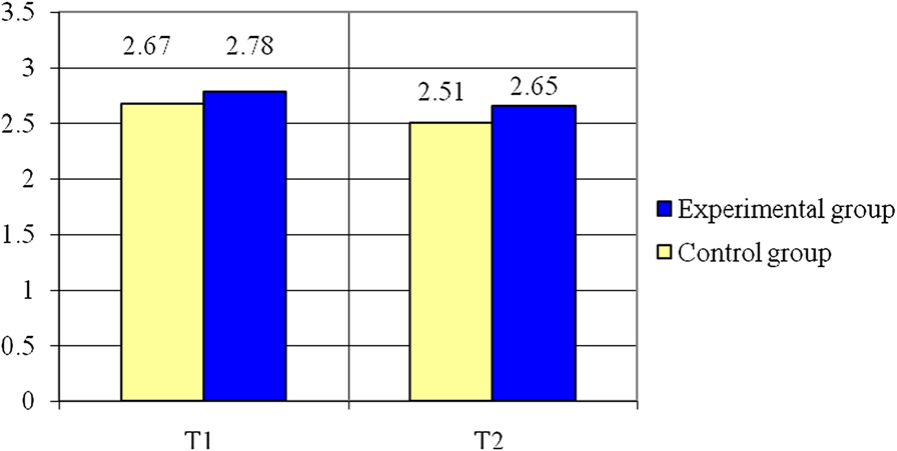
Successive jumps on steps (s) – averages.

The next test, Successive Jumps on Steps (see Table 2 as well as the Figure 7) indicates that the experimental group registers a decrease of 5.99% (0.16 s) to the final test as compared to the initial one and the control group registers a decrease of 4.68% (0.13 s). The variable is normally distributed (p > 0.05). Applying the Student test, it can be noticed that there are significant differences between the means of the two groups (t = 2.59, p < 0.015). The value of statistical power of the test (0.72) is considered acceptable.

**Figure 8:**
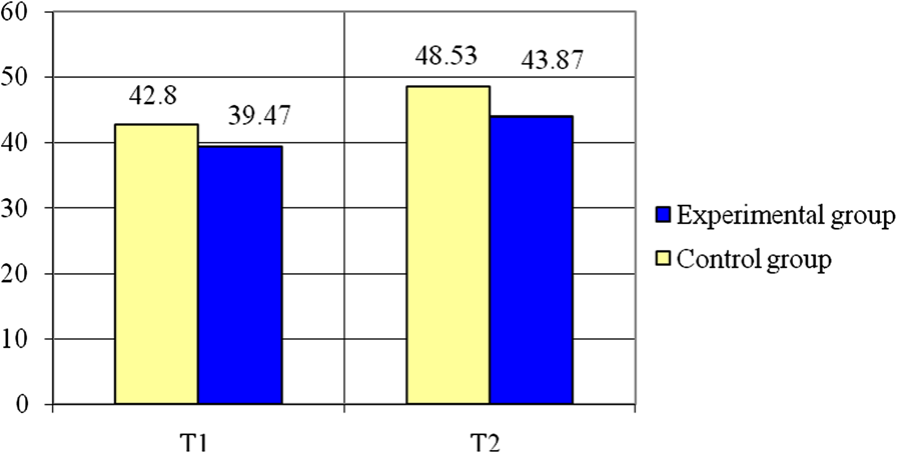
Remote ball hitting - dominant inferior limb – averages.

**Figure 9:**
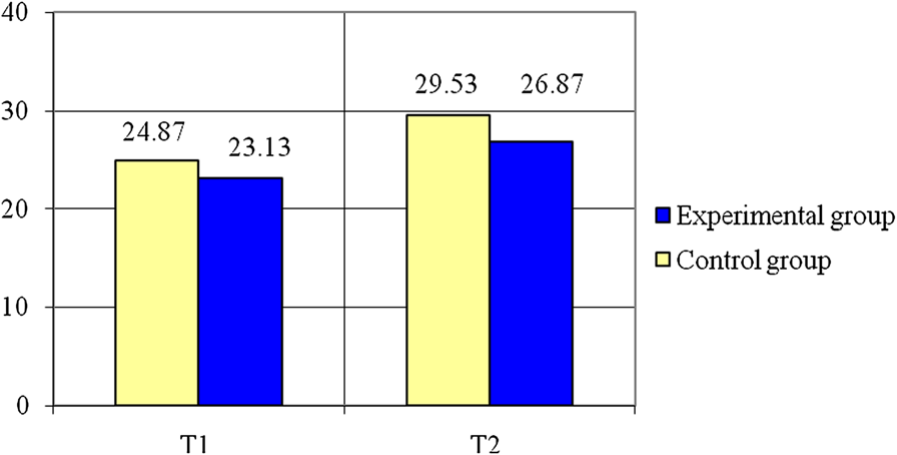
Remote ball hitting – non-dominant inferior limb – averages.

Regarding Remote Ball Hitting - (see Table 2 and Figures 8, 9) statistically significant differences of means were registered for both cases: for the dominant inferior limb (t = 2.25, p < 0.05) and for the non-dominant inferior limb (t = 2.21, p < 0.05), the experimental group having better results than the control group. The variables are normally distributed (p > 0.05). The power of the test (0.58 for the dominant inferior limb and 0.56 for the non-dominant inferior limb) conducts to a higher probability for correct decision than error.

The results and the statistic parameters help us to observe that the contraction time, Tc, registers a decrease on the right side, for group 1, of about 2.14% but, at the same time, an increase of about 17.43% for group 2. On the left side, Tc increases around 11.15% for group 2 and decreases around 1.72% for group 1. Comparing the evolution of Tc during the muscle training, one may observe that group 1 registers a Tc decrease, which is not the same for group 2.

Considering the displacement Dm, group 1 registers an important increase of 4.26% on the right side, while group 2 indicates a decrease in Dm of about 21.74%. When dealing with the left side, group 1 manifests an increase of 2.02% in Dm, while group 2 registers a decrease of about 15.52%.

One can assume that group 2 manifests an increase of muscle tone due to the concentric contraction [11] which represents the basis of the muscle training for this group of athletes. The results achieved by group 2 indicate an increase in Tc and a decrease in Dm, meaning a slow process of recruiting MU [1,12] and a low rate of Type I muscle fibres, while group 1 registers a decrease in Tc correlated to an increase in Dm, involving a high rate of Type II muscle fibres.

## Conclusions

These results are confirmed by the practical aspects of the evolution manifested by the subjects submitted to the study, using motor tests, which stand for the presence of a significant improvement of motor parameters, quantified through specific motor assessment tests. Increasing the distance when dealing with the standing long jump or the remote ball hitting tests, and reducing the time of the test consisting of jumping on steps, represent aspects meant to support the need for undertaking certain TMG investigations, allowing a right assessment of the muscle content, which is highly important when developing a sport activity, focused on the performance of an adapted muscle training involving the muscle fibers, transferred to the muscle area, considering the effort intensity and the targeted motor performances.

A high rate of Type II muscle fibres for group 1 involves an increase in knee stability.

This assessment method supports the presence of a muscle training program focused on the muscle fibres potential to convert from one type to another, under the circumstances of a specific muscle training program.

In conclusion, the measurement of muscle contraction parameters, following an electric stimulation of the muscle group, enables us to adjust the muscle training to the effort intensity [1].

We also consider that relying on the neuromuscular assessment we can succeed in conceiving an individualised muscle training program or a specific muscle training program for each position of the football player within his/her team.

The limitation of the study was that soccer players are from juniors and some of them have problem for support the electrical stimulation. Also another problem was the presence of joint injuries during the research and so this limits the players’ participation to training program. Another limitation is given by the methodological factors like sensor position relative to the muscle belly and electrodes placement, but we consider that we minimize this aspect.

Future of TMG consists in build the training and rehabilitation program. The coach has the possibility to know more about the functional muscle symmetry and to determine what differences exist between playing positions. After, he can design the session training. Much more the TMG results help the coach to establish the future training load and this is very important for prevent muscle injuries.

This non-invasive method can be use very easy, independent of voluntary muscle contraction and has a real usefulness by detect the contractile muscle proprieties within subject muscle group imbalances which are important for training and in the same time for rehabilitation and recovery protocols.

## Abbreviations

TMG: Tensiomyography; RF: Rectus femoris; RF-R: Rectus femoris right side; RF-L: Rectus femoris left side; MyHC: Myosin heavy chain; MU: Motor unit.

## Competing interests

The authors declare that have no competing interests.

## Authors’ contributions

LR- sports medicine and rehabilitation physician, professor, the design of the study, the neuromuscular assessment using tensiomyography, the management of the research. GC - trainer, assoc prof., the design of the muscle training and the implementation of this training program. MM - engineer, professor, specialist in biomechanics field; the design of the muscle training and the neuromuscular assessment. SC - engineer, assoc. prof., the statistical analyses. PFR - engineer, doctoral student/Phd candidate, the data collection and the statistical analyses. CD *-* lecturer and team coach, propose and apply the training program. NF-lecturer, participates to data collection and training program. All authors read and approved the final manuscript.
